# Supplementary material for: Multivariate Analysis of Amino Acids and Health Beneficial Properties of Cantaloupe Varieties Grown in Six Locations in the United States
Source: Plants (Basel). 2020 Aug 19;9(9):1058. doi: 10.3390/plants9091058 (PMC7570236; doi:10.3390/plants9091058)
Supplement: Supplementary file 1 [file plants-09-01058-s001.pdf]

Multivariate Analysis of Amino Acids and Health Beneficial Properties of Cantaloupe Varieties Grown in Six Locations in the United States

Jashbir Singh[1,3], Rita Metrani[1,3], Guddadarangavvanahally K. Jayaprakasha[1,3*], Kevin M. Crosby[1,3], Sadhana Ravishankar[2,3], and Bhimanagouda S. Patil[1,3*]

[1]Vegetable & Fruit Improvement Center, Department of Horticultural Sciences, Texas A&M University, 1500 Research Parkway, Suite A120, College Station, Texas 77845-2119, United States
[2]School of Animal and Comparative Biomedical Sciences, University of Arizona, 1117 E. Lowell Street, Tucson, AZ 85721, USA
[3]National Center of Excellence for Melon at the Vegetable and Fruit Improvement Center of Texas A&M University, College Station, TX 77845

*Corresponding authors

Bhimanagouda S. Patil

Tel.: +1 979 862 4521; fax: +1 979 862 4522

e-mail:b-patil@tamu.edu

G.K. Jayaprakasha

Tel.: +1 979 845 2743; fax: +1 979 862 4522

e-mail: gkjp@tamu.edu

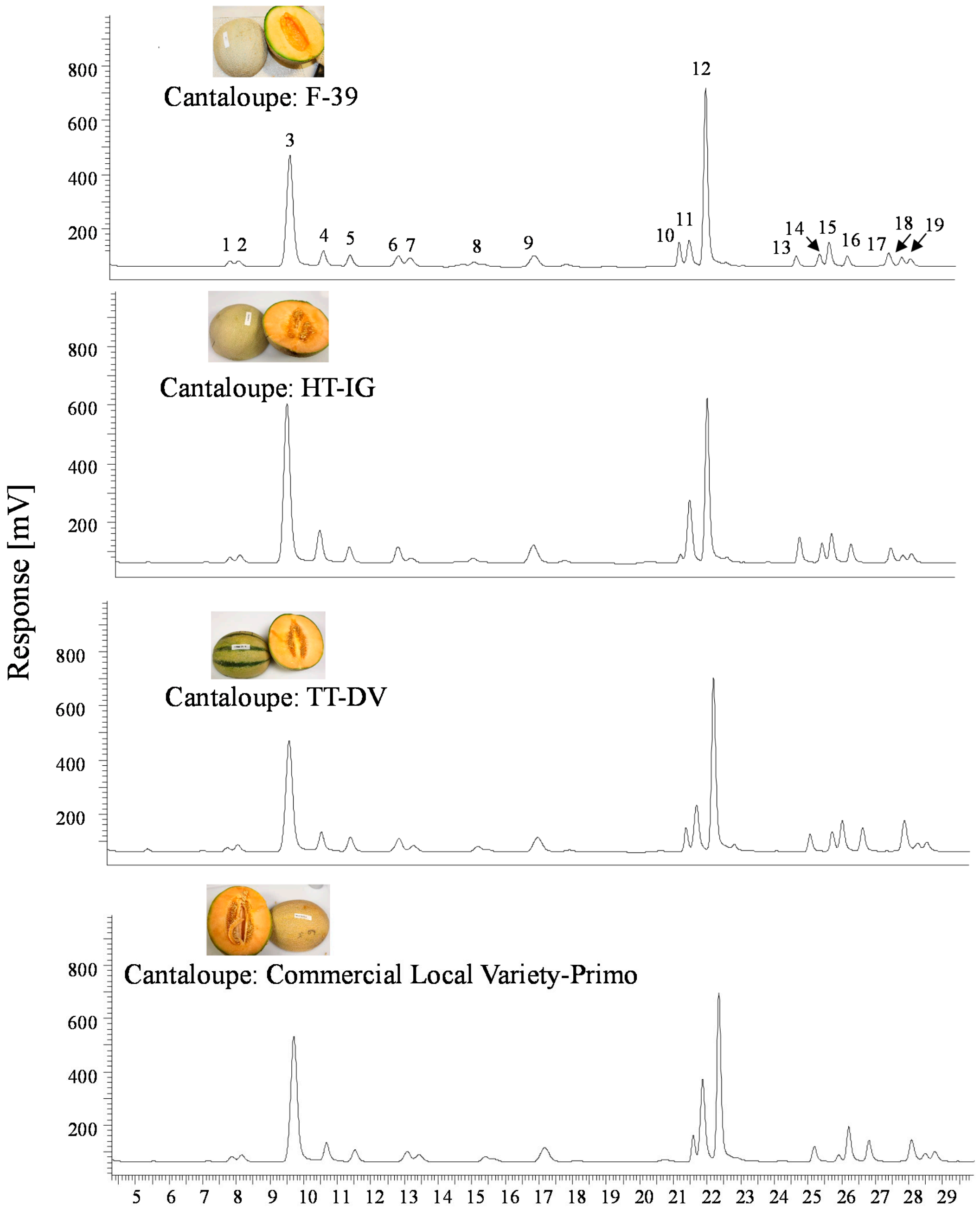

Figure S1. Comparative HPLC-FLD chromatograms of amino acids from different cantaloupe varieties grown in Texas, Uvalde. Cantaloupe varieties: Western Shipper (F-39), Harper-type Infinite Gold (HT-IG), Tuscan type Da Vinci (TT-DV) and commercial local varieties-Primo. Amino acids: 1) arginine; 2) asparagine; 3) glutamine; 4) citrulline; 5) serine; 6) aspartic acid; 7) hydroxy proline; 8) Threonine; 9) glycine; 10) β-alanine; 11) alanine; 12) γ-aminobutyric acid; 13) proline; 14) methionine; 15) valine; 16) tryptophan; 17) phenylalanine; 18) isoleucine; 19) leucine.

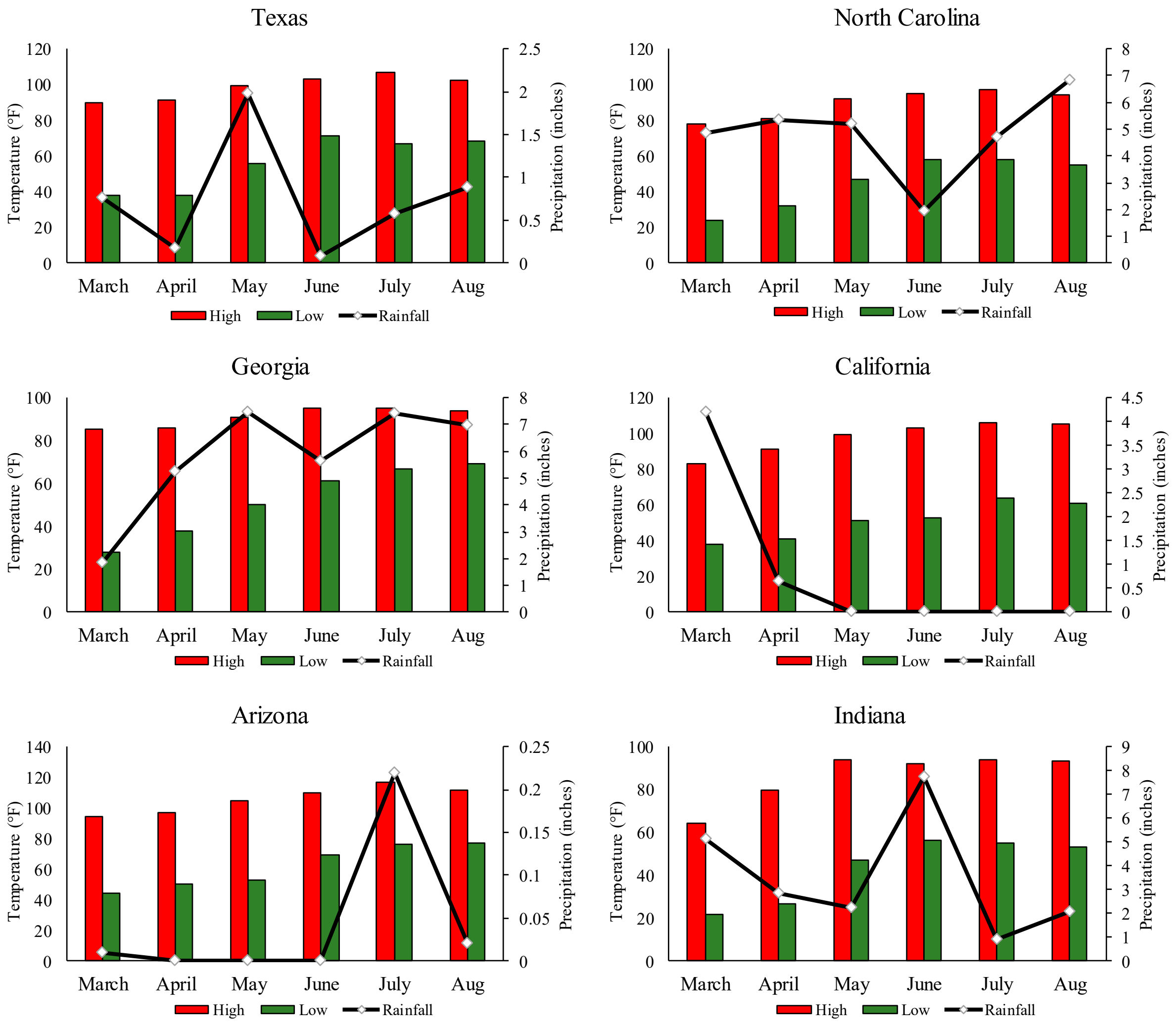

Figure S2. Precipitation (inches) and temperature (°F) of cantaloupe growing locations. Source: https://www.timeanddate.com/weather.

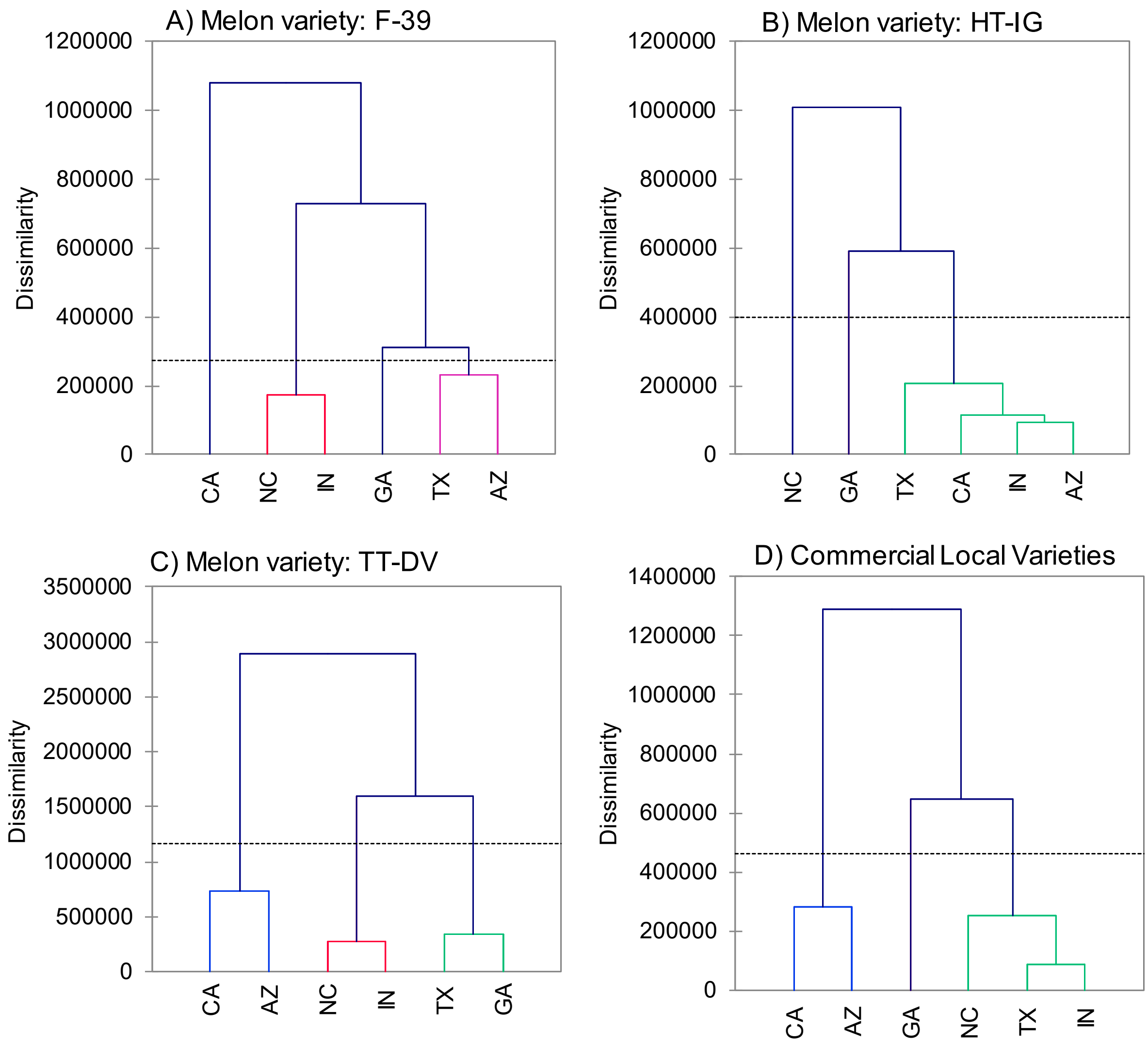

Figure S3. Hierarchical cluster analysis of melon varieties harvested from different locations (dendrograms using average linkage between groups). Texas (TX), Georgia (GA), North Carolina (NC), California (CA), Indiana (IN), and Arizona (AZ). Cantaloupe cultivars: Western Shipper (F-39), Harper-type Infinite Gold (HT-IG), Tuscan type Da Vinci (TT-DV) and commercial local varieties, Primo (PRI) from TX, Athena (ATH) from GA and NC, Alaniz gold (ALG) from AZ, Caribbean king (CAR) from CA and Cruiser (CRU) from IN.

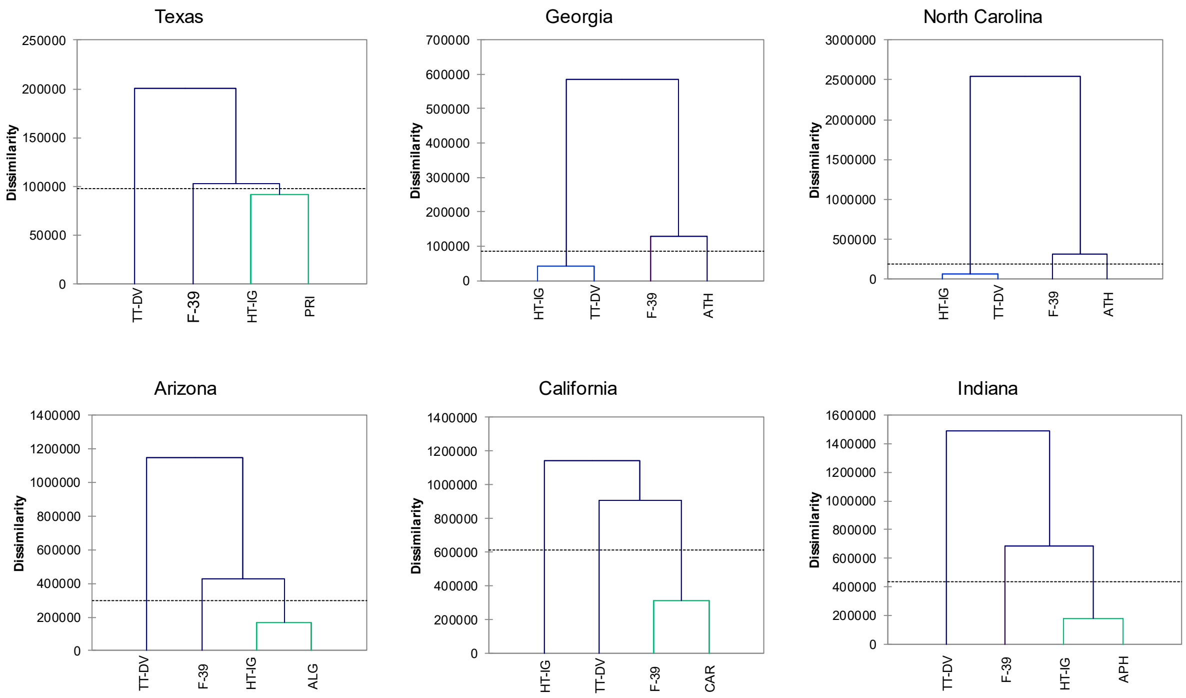

Figure S4. Hierarchical cluster analysis of four different melon varieties harvested from the same locations (dendrogram using average linkage between groups). Texas (TX), Georgia (GA), North Carolina (NC), California (CA), Indiana (IN), and Arizona (AZ). Cantaloupe cultivars: Western Shipper (F-39), Harper-type Infinite Gold (HT-IG), Tuscan type Da Vinci (TT-DV) and commercial local varieties, Primo (PRI) from TX, Athena (ATH) from GA and NC, Alaniz gold (ALG) from AZ, Caribbean king (CAR) from CA and Cruiser (CRU) from IN.

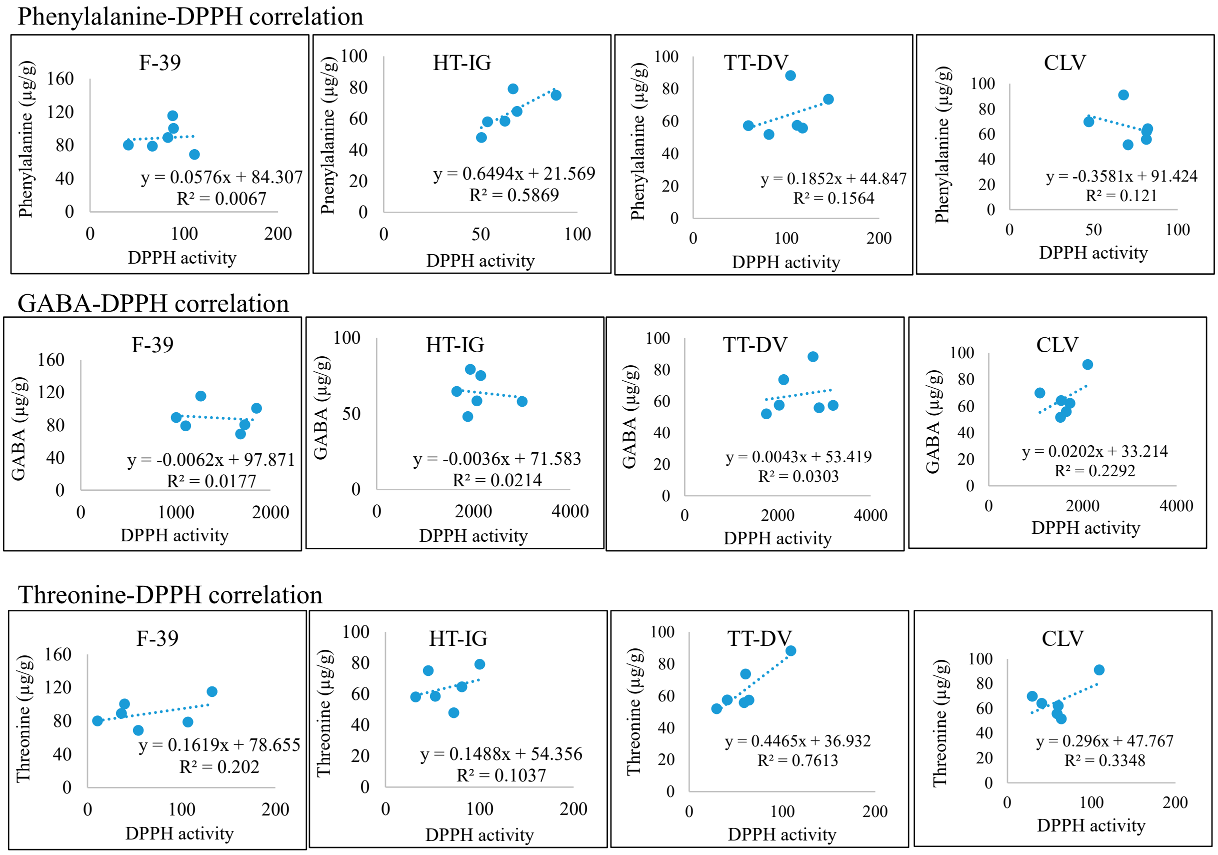

Figure S5. The relationship between selected amino acids and DPPH assay. Cantaloupe varieties: Western Shipper (F-39), Harper-type Infinite Gold (HT-IG), Tuscan type Da Vinci (TT-DV) and Commercial local varieties (CLV); Primo (PRI) from TX, Athena (ATH) from GA and NC, Alaniz gold (ALG) from AZ, Caribbean king (CAR) from CA and Cruiser (CRU) from IN.

.

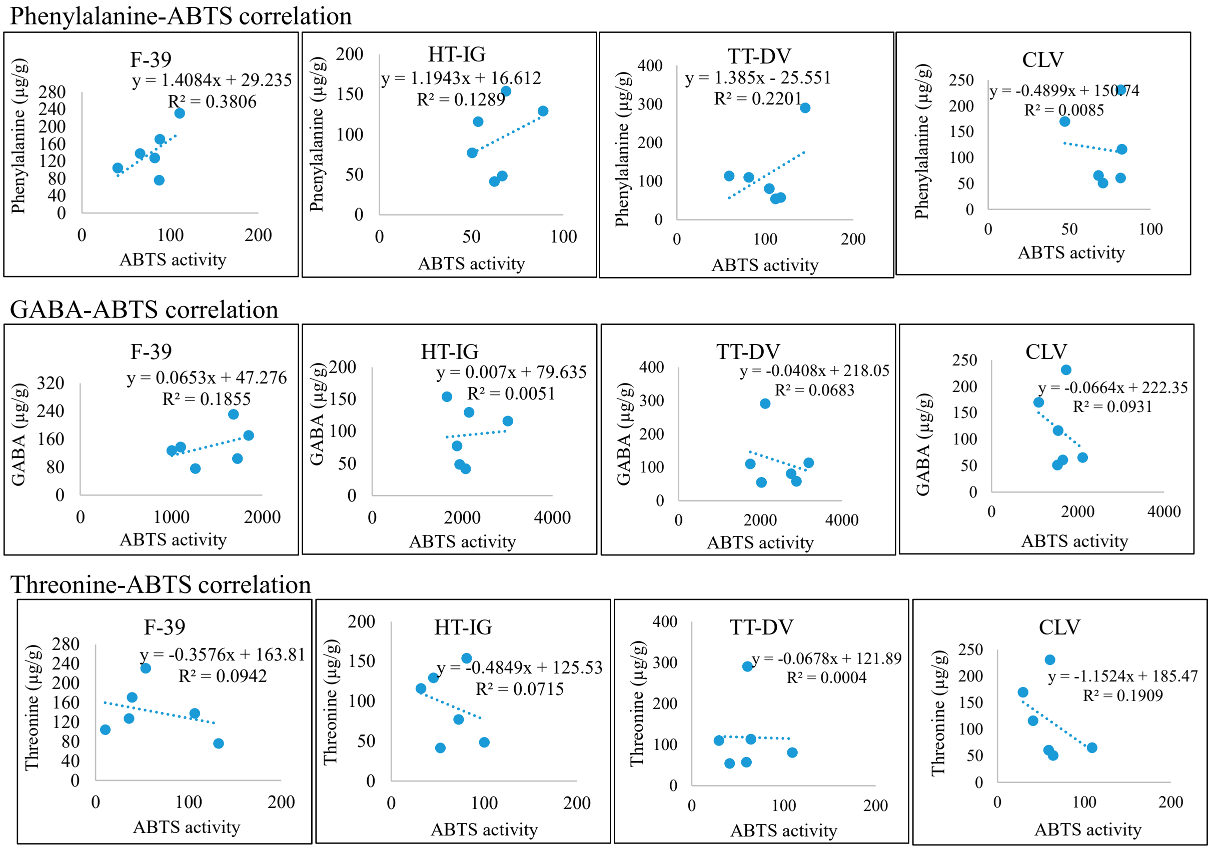

Figure S6. The relationship between selected amino acids and ABTS assay. Cantaloupe varieties: Western Shipper (F-39), Harper-type Infinite Gold (HT-IG), Tuscan type Da Vinci (TT-DV) and Commercial local varieties (CLV); Primo (PRI) from TX, Athena (ATH) from GA and NC, Alaniz gold (ALG) from AZ, Caribbean king (CAR) from CA and Cruiser (CRU) from IN.

Table S1. *p*-values of amino acids for different cantaloupe varieties.

Amino acids	F-39	HT-IG	TT-DV	Commercial local varieties
Arg	< 0.0001	< 0.0001	< 0.0001	< 0.0001
Asn	0.0002	0.0233	< 0.0001	< 0.0001
Gln	< 0.0001	0.0003	< 0.0001	< 0.0001
Cit	0.0001	< 0.0001	< 0.0001	0.0001
Ser	0.0003	0.0360	< 0.0001	< 0.0001
Asp	< 0.0001	0.1282	< 0.0001	< 0.0001
Hyp	< 0.0001	< 0.0001	0.8363	< 0.0001
Thr	< 0.0001	0.0281	0.0335	< 0.0001
Gly	0.0001	0.0130	< 0.0001	< 0.0001
β-Ala	< 0.0001	< 0.0001	< 0.0001	< 0.0001
Ala	0.0006	< 0.0001	< 0.0001	< 0.0001
GABA	< 0.0001	0.0040	< 0.0001	0.0190
Pro	0.0033	0.0033	< 0.0001	< 0.0001
Met	0.0010	0.0574	< 0.0001	< 0.0001
Val	< 0.0001	0.1401	< 0.0001	< 0.0001
Try	0.0317	< 0.0001	0.0291	< 0.0001
Phe	0.0001	0.0017	< 0.0001	0.0584
Ile	0.0140	0.4010	< 0.0001	0.0001
Leu	< 0.0001	0.0273	< 0.0001	< 0.0001

Cantaloupe cultivars, Western Shipper (F-39), Harper-type Infinite Gold (HT-IG), Tuscan type Da Vinci (TT-DV) and commercial local varieties, Primo (PRI) from Texas (TX), Athena (ATH) from Georgia (GA) and North Carolina (NC), Alaniz gold (ALG) from Arizona (AZ), Caribbean king (CAR) from California (CA) and Cruiser (CRU) from Indiana (IN). Amino acids: Arg: arginine; Asn: asparagine; Gln: glutamine; Cit: citrulline; Ser: serine; Asp: aspartic acid; Hyp: hydroxy proline; Thr: threonine; Gly: glycine; β-Ala: beta-alanine; Ala: alanine; GABA: γ-aminobutyric acid; Pro: proline; Met: methionine; Val: valine; Trp: tryptophan; Phe: phenylalanine; Ile: isoleucine; Leu: leucine.

Table S2. Pearson's correlation coefficients (r) of total phenolics and antioxidant activities in different cantaloupe varieties.

Variety	Compounds	Total Phenolics	DPPH	ABTS
F-39	Total Phenolics	1		
	DPPH	0.1337	1	
	ABTS	0.2392	0.4384	1
HT-IG	Total Phenolics	1		
	DPPH	0.4436	1	
	ABTS	0.3676	0.602	1
TT-DV	Total Phenolics	1		
	DPPH	0.4197	1	
	ABTS	0.2588	0.5474	1
Commercial Local Varieties	Total Phenolics	1		
	DPPH	0.1039	1	
	ABTS	0.3954	-0.438	1

Abbreviations: Western Shipper (F-39), Harper-type Infinite Gold (HT-IG), Tuscan type Da Vinci (TT-DV).

Table S3. Location and growing conditions for melon variety.

Location	Growing conditions
Indiana	Seeds of all the varieties were planted into 50-cell black seeding flats (T.O. Plastics, Clearwater, MN) on April 2018, using a peat-based potting media (Metro-Mix® 360, a mixture of sphagnum peat moss, coarse perlite, bark ash, starter fertilizer, and dolomite). Plants were transplanted to the field on May2018.<br>The soil type of the experimental field is sandy loam with 0.8 percent organic matter. Plants were grown in raised beds covered with a 4 ft wide black plastic mulch (Visqueen 4020). Drip tape with a 12-inch emitter spacing and flow rate of 0.22 gpm/100 ft was used for irrigation. Bed spacing and in-row spacing were 6 and 2.5 ft, respectively. Fertilizers at the rate of 250 lb/acre urea (46-0-0), 100 lb/acre potash (0-0-60), 100 lb/acre diammonium phosphate (18-46-0), 200 lb/acre pelletized lime, 100 lb/acre K-Mag granular (0-0-22-11-22), 7 lb/acre boron 14.3% and 10 lb/acre Zinc 10% LS were pre-plant broadcast applied. During transplanting, each plant received approximately one cup of starter fertilizer solution (Miracle-Gro, 4.7 grams per gallon water).
Georgia	Five week old transplants were planted on March 2018 in Tifton, GA. The soil was a Tifton loamy sand (~90% sand). Organic matter ranged from 0.4-0.8%.<br>Prior to planting 500 lb of 10-10-10 fertilizer (Rainbow, Agrium) was applied under plastic mulch, followed by a weekly fertigation program with 7-0-7 liquid fertilizer applied at a rate of 12 lb./acre of nitrogen until harvest. After transplant, water was daily applied at an irrigation depth of 0.2-in for a 20 days period to ensure plant establishment. Standard procedures for pest management in cantaloupes were followed with weekly funigicide and insecticide applications.
Texas	The experiment was conducted at the Texas AgriLife Research and Extension Center at Uvalde, TX (29°12’57.6’’N, 99°45’21.6’’W). Seeds of each cultivar were directly sown on raised beds covered with black plastic mulch. Beds were spaced 3.96 m (center to center). Standard commercial melon production practices in terms of fertilization and irrigation were followed. A weekly fertigation system was used to apply a seasonal N-P-K fertilizer rate of 87-20-85 lb./acre

California	The experiment was conducted at the University of California West Side Research and Extension Center in Fresno County (17353 W. Oakland Ave., Five Points, CA 93637at 36o20'14.5032"N, 120o06'42.5124"W). Each plot was 30 feet in length x a single 80-inch wide bed. Seeds of melon entries were sown into a Panoche clay loam soil.
Arizona	The experiment was conducted at Yuma Center of Excellence for Desert Agriculture (YCED). Soil sandy loam type was used for growing melon.
North Carolina	All plots in the study used black polyethylene plastic mulch and were fertigated with drip irrigation. A drip irrigation was applied throughout the growing season. A complete fertilizer (12-6-24 @ 400 lbs/ac) was applied. Fertigation with 7-0-7 liquid fertilizer was initiated 9 days after planting and applied weekly.
